# PP41 Effect Of SGLT2 Inhibitors On Renal Outcomes According To Glomerular Filtration Rate: Systematic Review Applying Clinical Benefit Thresholds

**DOI:** 10.1017/S0266462325102602

**Published:** 2025-12-29

**Authors:** Fernando Tortosa, Santiago Torales, Florencia Alcoba, Emanuel Medina

## Abstract

**Introduction:**

Effects of sodium-glucose transport protein 2 inhibitor (SGLT2i) on diabetes, cardiovascular, and renal outcomes have been shown in the evidence, partially disaggregating results according to glomerular filtration rate (GFR) subgroups, which limits the ability to identify relevant benefits. In addition, when specific renal outcomes were analyzed, no thresholds were established to define clinical relevance. A review of evidence by specific GFR levels for critical outcomes was proposed, as defined by clinical benefit thresholds.

**Methods:**

We conducted a systematic review using the PRISMA checklist, searching relevant databases for double-blind controlled trials of 1,000 or more patients with a duration longer than six months, SGLT2i use, and results disaggregated by GFR levels. Based on utilities, thresholds were expressed as minimum important detectable (MID) events of absolute effect reduction (AER) per 1,000 individuals for death (MID=10), GFR deterioration (MID=22), and composite renal outcome (MID=16). The Credibility of Effect Modification Analyses (ICEMAN) tool was used to assess effect modification credibility.

**Results:**

Thirteen studies were selected (90,403 patients, 75% diabetic, mean initial estimated GFR of 61 mL/minute per 1.73 m^2^).

- Mortality: GFR (<60): 22,816 patients, relative risk (RR) 0.91 (95% CI: 0.82, 1.01), AER 0.3 percent, null effect, high certainty; GFR (>60): 55,037 patients, RR 0.89 (95% CI: 0.83, 0.96), AER 1.5 percent, probable reduction, moderate certainty.
- GFR deterioration: GFR (30 to 45): 9,335 patients, RR 0.76 (95% Ci: 0.61, 0.95), AER 3.4 percent, slight reduction, moderate certainty; GFR (45 to 60): 9,245 patients, RR 0.54 (95% CI: 0.45, 0.66), AER 1.9 percent, small or null effect, high certainty; GFR (>60): 43,635 patients, RR 0.61 (95% CI: 0.48, 0.78), AER 0.8 percent, null effect, high certainty.
- Composite renal outcome: GFR (<60): 27,114 patients, RR 0.83 (95% CI: 0.63, 1.08), AER 1.5 percent, slight reduction, low certainty; GFR (>60): 60,422 patients, RR 0.70 (95% CI: 0.58, 0.86), AER 0.9 percent, minimal reduction, moderate certainty.

**Conclusions:**

SGLT2i may have relevant effects in patients with a GFR less than 60, with slight reductions in mortality rate and improvements in clinically important composite renal outcomes. When the GFR is greater than 60, the benefits are uncertain or not clinically relevant, underlining the importance of individually tailored treatments considering renal function for each patient.

